# A transcriptome analysis of two grapevine populations segregating for tendril phyllotaxy

**DOI:** 10.1038/hortres.2017.32

**Published:** 2017-07-12

**Authors:** Jie Arro, Jose Cuenca, Yingzhen Yang, Zhenchang Liang, Peter Cousins, Gan-Yuan Zhong

**Affiliations:** 1 USDA-Agricultural Research Service, Grape Genetics Research Unit, Geneva, NY 14456, USA; 2 Beijing Key Laboratory of Grape Science and Enology and Key Laboratory of Plant Resource, Institute of Botany, the Chinese Academy of Sciences, Beijing 100093, People’s Republic of China; 3 E&J Gallo Winery, Modesto, CA 95353, USA

## Abstract

The shoot structure of cultivated grapevine *Vitis vinifera* L. typically exhibits a three-node modular repetitive pattern, two sequential leaf-opposed tendrils followed by a tendril-free node. In this study, we investigated the molecular basis of this pattern by characterizing differentially expressed genes in 10 bulk samples of young tendril tissue from two grapevine populations showing segregation of mutant or wild-type shoot/tendril phyllotaxy. One population was the selfed progeny and the other one, an outcrossed progeny of a *Vitis* hybrid, ‘Roger’s Red’. We analyzed 13 375 expressed genes and carried out in-depth analyses of 324 of them, which were differentially expressed with a minimum of 1.5-fold changes between the mutant and wild-type bulk samples in both selfed and cross populations. A significant portion of these genes were direct *cis*-binding targets of 14 transcription factor families that were themselves differentially expressed. Network-based dependency analysis further revealed that most of the significantly rewired connections among the 10 most connected hub genes involved at least one transcription factor. TCP3 and MYB12, which were known important for plant-form development, were among these transcription factors. More importantly, TCP3 and MYB12 were found in this study to be involved in regulating the lignin gene *PRX52*, which is important to plant-form development. A further support evidence for the roles of TCP3-MYB12-PRX52 in contributing to tendril phyllotaxy was the findings of two other lignin-related genes uniquely expressed in the mutant phyllotaxy background.

## Introduction

Grapevine (*Vitis* spp.) is one of the oldest domesticated fruit crops in the world. Grown mostly for wine since antiquity, grapevine berries and their derivatives are also found in familiar consumer products such as table grapes, raisins, juice, jelly and dietary supplements due to recently known beneficial effect of its antioxidant, resveratrol.^1^ Understandably, until recently, the wine industry has driven a significant portion of the research on berry quality and wine-related traits such as color and juice compositional profile, as well as disease resistance and abiotic stress tolerance.^2^ In contrast, research needs in vine growth and development, and even yield have received much less attention with fewer number of research studies reported, even fewer than that related to vineyard management.^3^ Part of the reason that impedes a full understanding of grapevine developmental processes is the complex biology of grapevine itself.^4,5^ Grapevine takes more than a year to complete a growth cycle, one of the longest life cycles among the cultivated crops; its developmental stages involve many complex processes such as lateral bud formation, dormancy and burst, and obtaining reliable phenotypic data are challenging given its size, growing habits, viticulture practice and extensive interactions with external environments. In spite of these, some substantial knowledge has been gained about the vegetative and reproductive growth and development of grapevines, although the knowledge accumulation has rather been incremental.^3,6,7^

Unlike annual herbaceous plants, the growth and development of grapevine is unique and complex.^8^ The switch from juvenile to adult phase in Vitaceae, which encompass grapevines (*Vitis*) and ~900 member-species in other 15 genera,^9^ is signified by the appearance of the first tendril together with the switch from spiral to alternate leaf arrangement.^4^ From then on, growth proceeds with the shoot apical meristem (SAM) in the main branch’s apex continually giving rise to both leaf primordia and an uncommitted primordia called anlagen.^10,11^ Depending on the genetic and environmental cues, this uncommitted primordia can become one of the two homologous organs in Vitaceae, tendril or inflorescence. This feature is unique to Vitaceae. Any inflorescences formed in the vegetative phase of the first year, however, will undergo dormancy only to resume from the lateral buds in the following spring towards reproductive growth. This monopodial growth pattern, which spans a life cycle of at least 2 years characterized by indeterminate growth and lateral branching, defines the shoot architecture typical of Vitaceae.^12^ Thus, to some degree, growth and development in grapevine is inextricably linked to understanding its shoot organogenesis, shoot architecture and reproductive development.^4^

Except for a few, most species in the family Vitaceae possess tendrils and are known for their climbing habit.^5,13^ Tendrils are tactile string-like motile organs adapted for grappling and supporting the growing vine as it climbs atop canopies for maximum light interception in the wild. Tendrils are, as recognized by Darwin, adaptive morphological innovation in plants. Indeed, convergent adaptive evolution in other plants such as Fabaceae, Cucurbitaceae and Smilacaeae gave rise to tendrils from modified leaflets, shoots and stipules, respectively.^14^ In Vitaceae, however, tendril occupies a more significant biological role due to its close organogenetic relationship with inflorescences. Aside from their roles in movement and support, tendrils express inflorescence genes,^15–17^ and some of which were thought to control the miRNA-mediated phase change to flowering.^3^ Gerrath *et al.*^12^ recognized a modular three-node vegetative growth pattern in vine shoot growth and development, which in combination with presence and position of the latent buds, is a species-specific signature in Vitaceae. In the most widely cultivated *Vitis* species, *Vitis vinifera*, tendrils normally appear in repeating units of three nodes, opposite each leaf in two sequential nodes and then a skip on the third. However, in the North American wild *Vitis* species, *Vitis labrusca*, continuous presence of tendrils on nodes was observed.^18^

Some *Vitis* hybrids and cultivars were reported to show disruption in the regularity of the modular, three-node order of two successive tendrils followed by a skip. The *V. vinifera* var. ‘Grenache’ and ‘Syrah’ showed variable degrees of disrupted tendril phyllotaxy especially at the basal nodes,^19^ and that development in secondary axes were different depending on the position in the three-node module.^20^ Cousins *et al.*^21,22^ selfed a hybrid with disrupted tendril distribution pattern and also crossed it with a vine with normal tendril distribution. They observed segregation of the trait in both progeny populations. Taken together, this suggests that the three-node phyllotaxy is under genetic control. Cousins and Zhong^23^ proposed that the mutant type was likely controlled by two epistatic loci with supplementary dominant-gene action.

We report here an RNA-Seq differential expression analysis of bulk samples from two populations segregating for tendril phyllotaxy. Of the 13 375 expressed genes, we identified 324 differentially expressed genes (DEGs) in both populations. These DEGs include many transcription factor (TF) families involved in a myriad of growth and development processes. Our results shed light on the molecular processes involved in the tendril development, helping develop further understanding of the genetic control of reproductive traits and related plant architecture in grapevine.

## Materials and methods

### Plant material

Two segregating populations were used in this study (Table 1). One was derived from the cross of PC04206-36×Roger’s Red. The female parent, PC04206-36, is a nematode-resistant breeding selection from the USDA-Agricultural Research Service Grape Rootstock Improvement Program in Geneva, NY, USA.^23^ It shows a normal pattern of tendril phyllotaxy (a three-node pattern in which tendrils are present on the opposite sides of leaves in the first two sequential nodes but no tendril is on the third node). The male parent, Roger’s Red, is an ornamental vine valued for its deep red autumn foliage. Molecular marker data confirmed that Roger’s Red is a natural hybrid between *V. californica* and a red-fleshed *V. vinifera*, Alicante Bouchet.^24^ Roger’s Red has a high frequency of an interrupted or mutant pattern of tendril phyllotaxy (Cousins, unpublished data). This cross population, hereon referred to as ‘P65’, had a total of 175 progeny lines with 140 (80%) normal progenies and 35 (20%) mutant (Table 2). The other population used in this study, named as ‘P96’, was derived from selfed Roger’s Red. It had a total of 151 progeny lines with 64 (42%) of them showing a normal tendril distribution pattern and 87 (58%) a mutant pattern (Table 2).

### Phenotypic evaluation of tendril distribution patterns

Young seedling vines from germinated seeds were initially planted in 10 cm pots and then, when they reached to the stage with 4–5 leaves, transferred to 4-liter pots. Both crossed and selfed populations were planted about the same time in a greenhouse. The greenhouse had an average temperature of 26.7 °C and 14 h of light. All vines received routine fertilizer application and watering.

When the first tendril appeared, the node was marked with a label so that the node position could be easily followed later in a vine. Detailed observation of presence or absence of tendril was taken for each node in each vine. As a vine grew, more labels were used to keep track of the relative positions of nodes in the vine. Data from 18 or more nodes, after the first node with the tendril was observed, were taken and the tendril distribution pattern was determined accordingly. The phenotypes were classified into two categories. One was the wild-type phenotypes with two sequential tendrils followed by a skip: XXOXXO, where X represents presence of a tendril and O absence of a tendril. Conversely, the mutant-type phenotype represented tendril distribution patterns different from the wild-type, including XXOOX (a double skip), XXOOOX (a triple skip) or XXOOXOO (double–double skip). Although the disrupted mutant pattern was frequently observed in the proximal node, at the nodes 4–7, it was also observed at the distal nodes, such as at the nodes 10 and later.

### Tissue sampling and pooling

Determination of the tendril segregation pattern of a vine was usually completed when the vine grew to 18 or more nodes. On the basis of the observed patterns of tendril distribution, we grouped the vines into mutant and wild-type categories for each population. Then several bulks of samples were taken for both mutant and normal vines for each population. Each bulk samples consisted of several individual vines from which the two youngest tendril closest to the first open leaf in the apex were taken (Table 1). To maximize the bulk contrast of wild-type and mutant phenotypes, we selected those vines that showed similar disrupted phyllotactic patterns as much as possible in the mutant bulk samples and those vines which had no broken shoots in the wild-type bulk samples. In the end, a total of 21 P96 and 45 P65 progenies were pooled into 10 bulk samples for the construction of RNA-Seq libraries (Table 1). The bulk samples were frozen in liquid nitrogen and stored in −80C freezer until further processed.

### RNA-Seq library construction and sequencing

RNA-Seq libraries were constructed according to the protocols of Wang *et al.*^25^ and Zhong *et al.*^26^ Briefly, 20 μg total RNA was enriched for mRNA using oligo (dT) magnetic beads and then fragmented by incubating at 94 °C for 5 min in first strand synthesis buffer. From the fragmented mRNAs, first strand cDNA were synthesized with random hexamer-primer using SuperscriptIII Reverse Transcriptase (Invitrogen, Carlsbad, CA, USA). The resulting cDNAs were purified with Agencourt RNAClean XP beads (Beckman Coulter Genomics, Danvers, MA, USA), followed by end repair and dA-tailing (NEB, Ipswich, MA, USA), and then ligated with Y-shaped adapters using a concentrated T4 DNA ligase (Enzymatics, Beverly, MA, USA). The adaptor-ligated cDNAs were size-selected with Ampure XP beads (Beckman Coulter Genomics, Danvers, MA, USA) before PCR amplification with indexed primers. The RNA-Seq libraries amplified with indexed primers were sequenced using an Illumina HiSeq system at the Biotechnology Resource Center of Cornell University (Ithaca, NY, USA).

### Sequence data processing and differential expression analyses

Raw RNA-Seq sequence data in fastq format were cleaned by removing sequence artifacts such as adapter sequences, low quality trailing and leading reads using FastQC^27^ and Trimmomatic (Illumina, San Diego, CA, USA). The cleaned sequence reads were then individually aligned to the *Vitis* reference genome (12× *V. vinifera*, Phytozome ver 12) using the recommended alignment parameters for splice-aware transcriptome mapping as outlined in the Tophat2 workflow (http://ccb.jhu.edu/software/tophat/manual.shtml). Intermediary processing of the mapped reads (sam and bam files) such as read deduplication and read group renaming was done using Samtools,^28^ Bamtools and Picardtools.^29^

Once aligned, the count data required by edgeR were obtained as a sum of overlapping reads on the annotated gene feature (gff3 file) using HTseq.^30^ Differential gene expression analysis was done in R using the Bioconductor package, edge-R.^31^ The standard normalization step that adjusts differences in initial library sizes was done using the default trimmed means of median (TMM) method. TMM is a normalization of library reads based on the scaling of the library size that minimize the log-fold change.^31^ In addition, the ad-hoc minimum expression level for downstream analyses was set at 2 (count per millions of reads aligned) CPM for all libraries. DEGs were declared significant at the FDR ⩽0.05 and a 1.5-fold change.

### Functional and network co-expression analyses

To relate the biological significance of the discovered DEGs, gene ontology (GO) enrichment was conducted using Plant MetGenMAP.^32^ The resulting list of GO terms and their *P* values were reduced to a representative GO terms by clustering similar terms and projected into an MDS plot, as implemented by a web-based GO enrichment engine, Revigo.^33^

Transcription factor gene families as well as gene annotations were identified by cross-referencing two online plant functional databases: Plant MetGenMAP^32^ and PlantTFD ver. 4.^34^ Regulatory *cis* network prediction and TF enrichment tests were facilitated using the algorithm implemented by Plant RegMap.^34^

To assess changes in co-expression topology in the mutant and wild-type background, network-based analysis was carried out using the differential dependency network (DDN) algorithm^35^ as implemented in its Cytoscape-based version, knowledge-fused differential dependency network (KDDN),^36^ where the predicted *cis*-regulatory connections among the DEGs determined from Plant RegMap,^34^ was used as *a priori* information. Cytoscape^37^ was used to visualize the resulting co-expression network.

## Results

### Phenotypic observation of tendril distribution

Among 151 self-pollinated progeny seedling vines from the P96 population, we observed 87 vines with the mutant pattern of tendril distribution and 64 with the wild-type pattern (the normal tendril distribution pattern) (Table 2). The observations fit the expected 9 mutants: 7 wild-types segregation ratio for two loci with supplementary dominant interaction (*P* value=0.73) as proposed by Cousins and Zhong.^23^ Similarly, among 175 progeny lines derived from the cross population P65, we observed 35 mutant and 140 wild-type vines, also fitting the expected segregation pattern of 1 mutant: 3 wild-type (*P* value=0.12) (Table 2).

### Expression profile of DEGs

A total of 43.5 million 100-bp single-end reads were generated in this study, and 30.5 million (70–80%) were uniquely aligned to the reference genome (12× *V. vinifera*, Phytozome ver 12). These uniquely matched transcripts were used for downstream differential expression analyses as implemented by edgeR.^31^ On the basis of a preliminary exploratory analysis of the data, we set a minimum threshold of at least 2 CPM in all the RNA-Seq sample libraries for each gene to reduce potential false positives. As a result, a total of 13 375 expressed genes were retained. A multi-dimensional scale (MDS) analysis of these expressed genes revealed clear contrasts between mutant and wild phenotypes (*y*-axis), and between different genetic backgrounds (*x*-axis) (Supplementary Figure 1).

**Figure 1:**
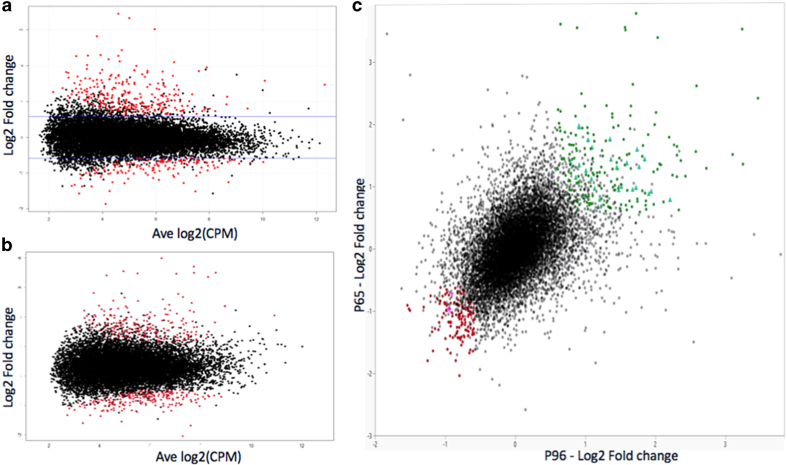
Expression profiles of differentially expressed genes (DEGs). (**a**) A log intensity ratio of expression versus average expression level (aka MA plot) of P96 depicting expression levels and fold-changes among 13 940 expressed genes; 349 DEGs highlighted in red. (**b**) MA plot of P65 depicting expression levels and fold-changes among 14 238 expressed genes; 467 DEGs highlighted in red. (**c**) Scatterplot of 13 350 expressed genes between P65 and P96. Highlighted are the 324 DEGs, composed of 201 upregulated (green dots) and 123 downregulated (red dots) DEGs, which were used for subsequent analyses.

In the self-pollinated population P96, a total of 13 940 expressed genes were detected, and 349 of them (2.6%) were significantly differentially expressed. Among these 349 DEGs, 257 and 92 were up- and downregulated, respectively (Figure 1a). The upregulated DEGs had larger fold-change (~1× to ~4× log2 FC) than the downregulated DEGs (~0.5× to ~2× log2 FC). In the cross population P65, a total of 14 238 expressed genes were detected, and 467 of them were differentially expressed (3.4%). Among these 467 genes, 227 and 240 were up- and downregulated, respectively (Figure 1b). Similar to P96, the upregulated DEGs had larger fold-changes ranging from ~1× to ~4× log2 FC, whereas the downregulated DEGs were between ~0.5× and ~2× log2 FC. It was noted that increasing significance stringency from FDR ⩽0.05 to FDR ⩽0.01 retained about half of the DEGs in the self-pollinated population (177 from 349 genes) and about a third in the cross population (163 from 467). Most of the retained genes were of higher expression levels.

### Expression correlation of DEGs between P65 and P96

A total of 13 375 expressed genes were detected in both P65 and P96 populations. A moderate positive correlation (*r*=0.46) between P65 and P96 was observed in the pairwise scatterplot of these genes based on their average fold-changes (Figure 1c). We further filtered the DEGs and focused our subsequent analysis on a subset of 324 DEGs, which had conforming expression profiles of at least 1.5-fold change in both populations and were significant at FDR ⩽0.05 in at least one of the populations. This subset, composed of 201 upregulated and 123 downregulated genes, had a correlation coefficient *r*=0.85 of fold-changes of expression between the two populations.

### Uniquely expressed genes

In the DEG analysis, we set a minimum expression level for a given gene at 2 CPM in all 10 libraries. Genes that were detected only in the wild or mutant libraries would be filtered out. We examined these excluded genes. We discovered four such genes that showed an average up- or downregulated expression level of ~2 CPM or more in a wild or mutant type with no reads detected in its counterpart.

Three genes were upregulated in the mutant background. The first one is a transcription factor (TF) bearing the AP2/ERF domain (GSVIVT01036388001) belonging to the integrase-type DNA-binding superfamily protein. This gene is homologous to *Arabidopsis*’ ERF22, which encodes a DREB protein involved in embryogenesis.^38^ The other two upregulated genes were related to secondary metabolite synthesis, with one (GSVIVT01015165001, UDP-glycosyltransferase superfamily protein) found in the flavonol pathway, and the other (GSVIVT01009107001, PRX52) in the matairesinol biosynthesis belonging to the lignin biosynthesis pathway. The only downregulated gene, which had no aligned transcript in the mutant library, was annotated as laccase (GSVIVT01013693001), another gene in the lignin biosynthesis pathway. Lacasse is homologous to *Arabidopsis*’ transparent testa 10 (TT10) involved in seed germination, root elongation and lignin degradation in the seed coat.^39^

### Functional analyses of 324 DEGs

Using Plant MetGenMap’s gene ontology database,^32^ 191 of the 324 DEGs (58%) were matched with GO annotations. The larger GO biological processes among the DEGs were ‘Cellular process’, ‘Response to stress’, ‘Metabolic process’, ‘Transport’, ‘Transcription’ and ‘Response to stimuli’. Subsequent GO enrichment analyses revealed that at a conservative cut-off threshold (FDR <0.10), 19 biological processes were enriched. As determined by using Revigo,^33^ these 19 biological processes grouped into four semantically similar biological processes: ‘Response to biotic and abiotic stimuli’, ‘Plant signaling’, ‘Plant transport’ and ‘Transcriptional regulation and cell communication’ (Table 3; Supplementary Figure 2). Although there was a large overlap among the member genes in these four clusters, each cluster was semantically distinct, defined by the frequency and identity of its member genes.

Cluster I roughly describes response to stimuli processes, having GO terms ‘Response to chitin’, ‘Response to carbohydrate’ and several stimuli-induced plant responses (Table 3). This cluster is comprised of 97 DEGs, majority of which were highly expressed and stress-inducible (Supplementary Table 1). Asparagine synthase (GSVIVT01024713001), a key gene in nitrogen assimilation and translocation^40^ and a stress-response gene in microbial infections like *Xanthomonas*,^41^ was the most upregulated gene among the 324 DEGs, with an average of fold of change at 3.4 log2 FC, and about twice the average positive fold-change of cluster I. On the other hand, cyclin B1–2 (GSVIVT01032782001), a gene that encodes a kinase-activating protein important to mitosis and cell cycle-related growth responses^42^ and triggered by many stress conditions such as high salinity,^43^ was one of the most downregulated gene with the fold of change at an average of 1.4 log2 FC. Majority of the DEGs in this cluster, especially those with large expression changes such as cytochrome P450 (GSVIVT01008261001), lysine-specific demethylase 3B (GSVIVT01026208001) and defensin protein (GSVIVT01010274001), were from different metabolic pathways but were directly or indirectly activated by biotic and abiotic stresses.^44–46^ Cluster I also includes 27 of the 30 differentially expressed TFs, 15 of which belong to the known major stress-response TFs gene families AP2/ERF, NAC and WRKY. More interestingly, closer scrutiny of their binding sites and targets, as determined using Plant RegMap,^34^ revealed that about half of the DEGs (37 out of 70) in this cluster were the direct downstream targets of these TFs. Included in the prominently upregulated targets (>1.7 log2 FC) were dehydrin gene 1 (GSVIVT01018878001), a target of AP2/ERF; peroxidase gene (GSVIVT01009106001), a target of bHLH and TCP; and protein TIFY 5A (GSVIVT01021514001), a target of NAC. Among the prominently downregulated (>1.2 log2 FC) targets were syntaxin gene (GSVIVT01035559001), a target of WRKY; thiamine thiazole synthase (GSVIVT01012636001), a target of AP2/ERF; and lysine-specific demethylase 3B (GSVIVT01026208001), a target of NAC. The aforementioned target genes were known for their role in stress conditions.^47–49^ Thus, taken together, cluster I, which corresponded to ‘Response to stimuli’, was mainly comprised of biotic and abiotic stress-inducible genes and their transcriptional regulators.

Cluster II is composed of four symantically similar GO terms related to ‘Plant signaling’ (Table 3; Supplementary Figure 2). It is composed of 19 DEGs, 15 of which were TFs, whereas three of the remaining four DEGs were downstream targets to WRKY and TCP gene families (Supplementary Table 1). The WRKY-targeted gene families include the downregulated plant signaling gene syntaxin^50^ (GSVIVT01035559001) and ABC transporter genes^51^ (GSVIVT01016999001), whereas the TCP-targeted genes included respiratory burst oxidase homolog (GSVIVT01019429001), a member of the redox-sensitive signaling.^52,53^ Cluster III corresponds to ‘Transport’ among the enriched biological processes, with two aquaporin homologs (GSVIVT01025038001, GSVIVT01016615001 ) that primarily mediate plant water transport activated during drought-stress conditions and ripening^54,55^ (Supplementary Table 1).

Cluster IV is a subset of 41 DEGs related to ‘Transcriptional regulation and cell communication’ processes (Table 3; Supplementary Figure 2). The upregulated genes in this cluster were mostly involved in stress-response mechanisms such as the cationic amino acid transporter (GSVIVT01034656001), a gene belonging to choline transporters activated during root-knot nematode infection,^56^ as well as MYC 2 (GSVIVT01013156001), a bHLH domain-carrying member of MYC-related gene family whose many biological roles include interaction with Jasmonate-zim domain (JAZ) to elicit drought tolerance in plants.^57^ On the contrary, the downregulated genes seemed to reflect more developmental regulatory genes. For example, the pseudo-response regulator gene (GSVIVT01032644001) was associated with circadian clock regulation, as well as phytochrome-dependent transduction,^58^ whereas genes such as RNA-binding gene (GSVIVT01009045001), neuroguidin gene (GSVIVT01009200001) and endoribonuclease dicer-like protein encoding gene (GSVIVT01027460001) were genes broadly related to post-transcriptional gene regulation.^59^ Similar to the previous clusters, cluster IV includes TFs and their downstream targets (Supplementary Table 1). Notable upregulated target genes among the 26 TFs in cluster IV were protein TIFY 5A (GSVIVT01021514001, a NAC downstream target), GID1, an important GA receptor gene (GSVIVT01011037001; a AP2/ERF downstream target) and putative-ubiquitin conjugating enzyme (GSVIVT01034196001, a TCP downstream target). Protein TIFY is a Jasmonate-ZIM domain protein 8 homolog belonging to the JAZ protein family and a noted key gene in reproductive developmental processes.^49^ GID1 is part of the GA pathway, a key component GA:GID1 complex bound by DELLA in maintaining the critical GA homeostasis.^60^ On the other hand, among the highly downregulated targets was lysis-specific demethylase 3B gene (GSVIVT01026208001; a NAC target). Its homolog in *Arabidopsis*, IBM1, was reported to mediate histone methylation processes involved in arrested flower and pollen development.^61^ Another important downregulated gene in this cluster is MYB12, a flavonoid biosynthesis activator.^62^ In grapevine, the secondary metabolites, such as flavonols, flavonoids and anthocyanins, have major roles in plant defense response^63^ in addition to their roles in wine-related quality traits.^64^ Apparently, this cluster of ‘Transcriptional regulation and cell communication’ biological processes includes the genes controlling developmental plant hormones, mainly GA and ABA, as well as secondary metabolites such as flavonoids, and even methylation-mediated regulations that involves dicer and RNA-binding protein. A TCP-domain TF, TCP3, was only found in this cluster but not in the others, suggesting that the molecular mechanism attributed to TCPs—plant form and structure—might be a significant part that defines an enriched transcription regulation and cell communication processes found in tendril phyllotaxy.

Unfortunately, only about 60% of the 324 DEGs could be accounted for in these GO enrichment analyses. Nevertheless, the analyses revealed that tendril/shoot phyllotaxy was intricately connected with the plant’s regulatory control for responses to internal and external stimuli, a large part of which might be, as shown in the enriched gene sets, mediated through *cis*-regulation.

### Dependency network analysis

To examine possible transcriptome-wide rewiring among the 324 DEGs, we carried out a network-based expression analysis using DDN. DDN examines changes in co-expression topology between two conditions (in this case, mutant and wild-type phenotypes) using a network-learning algorithm, to detect selectively activated or deactivated regulatory mechanisms. We used an enhanced version, KDDN.^36^ KDDN allows users to incorporate established biological information such as the pairwise *cis*-regulatory connections obtained from Plant RegMap^34^ as *a priori* knowledge in dependency network construction.

To examine the most plausible and relevant transcriptome network rewiring associated with tendril phyllotaxy from the complex KDDN-generated dependency network, we extracted the ten most connected (hub) genes and the first-degree connections around them. The extracted subnetwork consists of a total of 96 DE genes (Supplementary Figure 3). Hub genes are points of interest in expression networks because they, and connections around them, are likely to correspond to relevant biological regulatory roles in the proposed network.^65^

**Figure 2:**
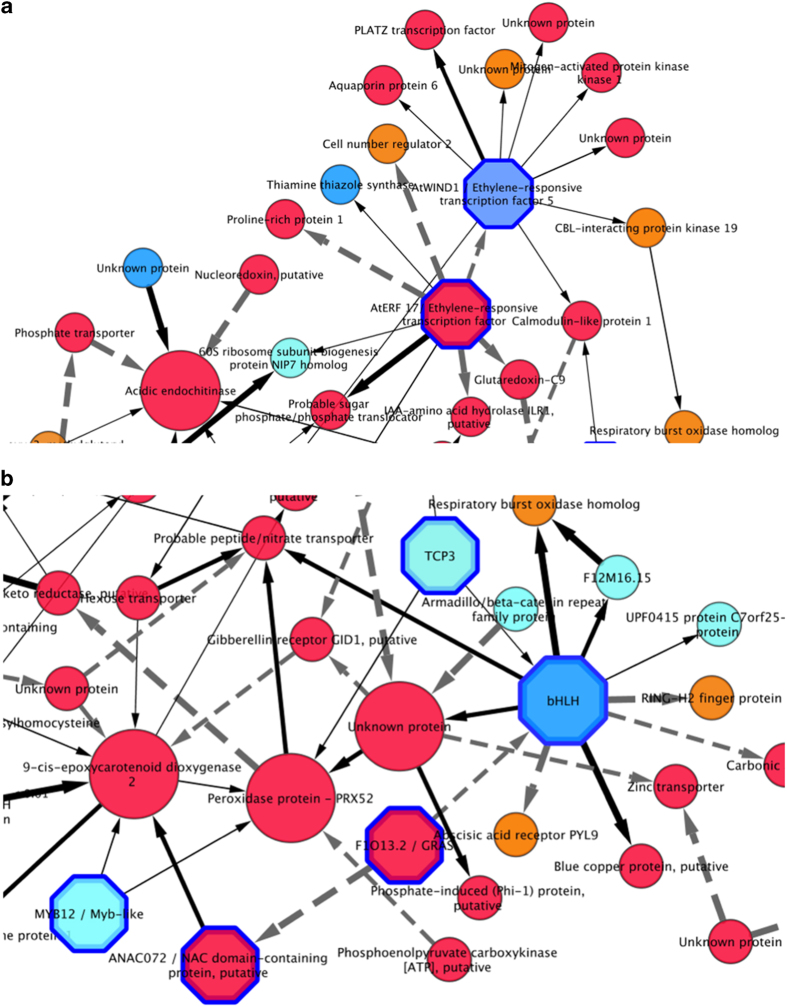
(**a**) AtERF17 and AtWIND1 (AP2/ERF) hub genes and their immediate connections in KDDN-generated dependency network. Orange and red nodes are moderately to highly upregulated genes. Light blue to dark blue nodes are moderately to highly downregulated genes. Octagon-shaped nodes are transcription factors. Connecting lines represent directional co-dependency in expression, with thicker lines indicate highly significant (*P*_val_ <0.01) connections in the mutant background, whereas broken lines indicate significantly deactivated connections in the mutant background. The size of a node is proportional to the number of connecting lines involved. (**b**) TCP3 and MYB12 relevant connections in the KDDN-generated dependency network among the 10 most connected hub genes. Orange and red nodes are moderately to highly upregulated genes. Light blue to dark blue nodes are moderately to highly downregulated genes. Octagon-shaped nodes are transcription factors. Connecting lines represent directional co-dependency in expression, with thicker lines indicate highly significant (*P*_val _<0.01) connections in the mutant background, whereas broken lines indicate significantly deactivated connections in the mutant background. The size of a node is proportional to the number of connecting lines involved.

Table 4 listed the hub genes identified from the dependency network analysis of the 324 DEGs. Most of these hub genes have known regulatory roles in plant stress responses. For example, two of the hub genes in Table 4 were related to the AP2/ERF gene family, which mediates developmental processes such as flower development,^66^ as well abiotic stress responses such as drought, high salinity and extreme temperatures.^55^ The first listed AP2/ERF hub gene, AtWind1, is a noted TF mediating callus formation during wound injury.^67^ It has 10 connections in the network, including genes in the signaling cascade pathway MAPK and calmodulin-binding protein kinase (Table 4; Figure 2a). As further revealed by the KDDN-generated dependency network, AtWind1 had a significantly strong connection (FDR <0.01) in the mutant background with PLATZ TF (Figure 2a), another plant-specific TF involved in cell differentiation.^68^ The other listed AP2/ERF hub gene is ERF17 (Figure 2a), a homolog of *Arabidopsis*’ AtERF17. The dependency network suggested that the ERF17 had connections with genes having wide biological roles such as transport (hexose transporter, GSVIVT01017937001; plant lipid transfer proteins, GSVIVT01015895001), hormone balance (IAA-amino acid hydrolase, GSVIVT01008852001) and reactive oxygen species (ROS)-mediated defense response (Roxy19, GSVIVT01021124001). Interestingly, the signal transducer Roxy19 belongs to glutaredoxin (GRX) gene family, a group downstream to TCP gene family regulation,^69^ which has been found related to shoot phyllotaxy in maize^70^ and petal formation in *Arabidopsis*.^71^ In addition, ERF17 belongs to the subclade of AP2/ERF gene family that readily interacts with GRAS TFs.^72^

Another hub gene listed in Table 4 is an RNA-binding homolog, one of the genes in the subset of enriched GO biological process ‘Plant regulation and cell communication’. Although its molecular function has yet to be fully elucidated, this RNA-binding gene is believed to primarily have a role in post-transcriptional gene regulation.^73^ Our KDDN network analysis suggested a significant connection in the mutant background with a calcium kinase 1-related gene (GSVIVT01022606001), a gene that phosphorylates phenylalanine ammonia lyase (PAL) and serves as a key enzyme in pathogen defense^74^ (Supplementary Figure 3). In addition, the RNA-binding hub gene’s connection was deactivated to a putative regulatory connections of NAC002 (GSVIVT01008839001), a TF active in abiotic stresses and pathogen infection response^75^ (Supplementary Figure 3). Taken together, the proposed dependency network emanating from the RNA-binding gene in this study re-confirmed the possible role of post-transcriptional regulation in plant stress response^76^ and/or shoot architecture.^77^

Perhaps the more interesting hub gene is the peroxidase gene (GSVIVT01030219001). Its *Arabidopsis* homolog, PRX52, is a major lignin and secondary cell wall biosynthesis gene especially in the stem and xylem vessels; loss-of-function mutants for this gene in *Arabidopsis* showed 70–80% reduction in lignin content, especially the syringyl lignin.^78^ It was proposed that it is one of the points of regulatory control in ABA-mediated defense responses during bacterial, fungal and insect attack.^47^ In our proposed KDDN network where it had seven connections (Table 4), PRX52 had a significant connection (*P*_val_ <0.01) in the mutant background with a glucosinate transporter 2 (GSVIVT01008072001), a member of the nitrate transporter1/peptide transporter family (NPF) transporters (Figure 2b). NPF were recently reported as critical carriers of GA and ABA hormone in grapevine.^79,80^ In addition, PRX52’s connection was deactivated in the mutant background with a carboxykinase gene (GSVIVT01005596001), which encodes a GTP protein in the gluconeogenesis pathway, which in turn is central to G-protein-mediated signal transduction in plant immunity.^81^ Interestingly, although the KDDN connections were not significantly rewired, PRX52 was connected to two downregulated TFs that have key regulatory roles in plant form and defense responses (Figure 2b). Our dependency network correctly depicted PRX52 as one of the downstream targets of TCP3, a class II CIN-TCP TF. The class II TCP transcription factor that bears the CINCINATA motif such as TCP3^69^ has been shown to regulate the morphogenesis of shoot lateral organs, as well as correct petal and stamen development^82,83^ and defense responses.^84^ It is highly possible that PRX52’s role in lignification might be related to TCP3’s wider role in plant form and stress response. PRX52 was also connected to a flavonol-specific activator, MYB12.^85^ The flavonol and flavonoid pathways produce secondary metabolites and share the phenylpropanoid pathway with lignin biosynthesis, pathways that have been implicated in plant defense responses in grapevine.^86^

### Regulatory connection among TFs and their target genes

The considerable proportion of TFs and their targets among the sets of enriched biological processes earlier examined suggested a substantial role of *cis*-regulation in the discovered DEGs. Indeed, based on the Plant RegMap’s *cis*-regulatory database,^34^ 21 TFs (out of the 30) and their predicted targets accounted for about 35% of the observed DEGs (123 of the 324 DEGs). A formal enrichment test revealed that nine genes belonging to five TF families had significantly over-represented targets: AP2/ERF, WRKY, NAC, bHLH and TCP (Table 5). As was earlier noted, the AP2/ERF domain family has the highest number of downstream targets, with three AP2/ERF genes accounting for ~28% of the total DEGs (Table 5). With 40 targets, ERF17 (GSVIVT01015037001) dominated the possible *cis*-binding interactions in the observed DEGs, perhaps due to its cross interactions with other TFs. ERF17 belongs to the A-5 DREB subfamily that interacts with other DREB subfamilies in regulating cold and high-salinity tolerance.^55^ In addition to its putative role in organic acid accumulation,^87^ ERF17 is also classified to belong to the gibberellin-related clade of the AP2/ERFs family because of its tendency to interact readily with DELLA of the gibberellin pathway.^72^ With 23 downstream targets, the second ranking gene belonging to the AP2/ERF gene family is WIND1 (GSVIVT01009007001), a noted TF in cell differentiation.^67^ The third AP2/ERF gene (GSVIVT01021098001) is a homolog of ERF09, an AP2/ERF known for its role in pathogen-related defense responses.^66^ It is noteworthy to recognize that both ERF17 and WIND1 were also the most connected hub genes in the expression topology-driven dependency network (Table 4; Supplementary Figure 3), reinforcing the substantial role of *cis*-regulation among co-expression profiles of the 324 DEGs.

A bHLH homeodomain TF (GSVIVT01018165001) was also significantly enriched with downstream targets of 20 DEGs (Table 5). This particular bHLH TF is homologous to UNE10, a regulator active during seed fertilization in *Arabidopsis*.^88^ The bHLH gene family are also one of the key components in the ternary complex of TFs (MYB-bHLH-WD40) required for the initiation of the anthocyanins and proanthocyanidins.^89^ Interestingly, it was also found to be one of the major hub genes in the dependency network as revealed in this study (Table 4; Supplementary Figure 3).

NACs are largely involved in the ABA-dependent stress signaling pathway. Two NAC TFs were found significantly enriched in this study. The first one was homologous to AtAF1, which in *Arabidopsis* was mediated via the ROS signal transduction pathway in responding to many abiotic stresses and pathogen infections stimuli.^75^ The second NAC gene (GSVIVT01014403001) was a homolog of RD26, which was noted to be insensitive to jasmonic acid-related stress signaling.^90^

TCP3 was also among the enriched with a predicted downstream target of 24 DEGs. A class II CIN-TCP, TCP3 is recognized a key regulatory control of shoot morphogenesis through negative regulation of the boundary-specific genes through miRNA induction.^83^

### Tendril and flower-identity genes

In many ways, the complex regulatory dynamics and the relatively large number of development-associated TFs uncovered in this study are typical of actively developing organs such as a tendril. The grapevine tendrils, however, are homologous to inflorescences and were reported to express flower-identity genes such as AP1, FUL, FT and LFY.^7,13,15^ We observed that all the homologs of the floral-identity genes AP1, FUL, FT were expressed at varying expression levels in our experiment. However, they were not differentially expressed between mutant and wild-types. We also confirmed that the LFY homolog was not expressed in the tendril transcriptome, which was in agreement with the observation that the gene seemed expressed only in grapevine inflorescence and not in tendril.^7,13^

## Discussion

Unique to grapevine shoot growth is the simultaneous differentiation of reproductive and vegetative organs in the same meristematic cells in shoot apex, and a number of studies have contributed to our understanding of the underlying biology of this phenomenon.^4,91,92^ Although the growth pattern of vine shoot development remains largely a point of conjecture as sympodial or monopodial growth,^18,93^ the resulting shoot phyllotaxy has been found more or less constant within given taxonomic groups and can be used to differentiate them in Vitaceae.^18^ The shoot phyllotaxy in vines is presumably under genetic control, but experimental evidence to support the claim is scarce. Recently, Cousins *et al.*^22^ reported that seedling populations derived from self-pollination of interspecific *Vitis* hybrid cultivars and from hybridization of *Vitis* hybrid cultivars, showed a high incidence of abnormal or mutant tendril distribution; and a subsequent study of the tendril segregation patterns from the self-pollinated and crossed progeny derived from the hybrid suggested that the mutant type was likely controlled by two epistatic loci^23^ with a supplementary dominant-gene action. In this study, we observed similar segregation patterns from the same sets of populations reported by Cousins and Zhong^23^ and confirmed that the hypothesis they proposed offered the most satisfactory explanation of the genetic control of the tendril phyllotaxy or distribution patterns in the *Vitis* species. Further validation of the hypothesis can be carried out, for example, by mapping QTL loci controlling the tendril distribution patterns using appropriate mapping populations such as those used in the present study.

To elucidate the genetic and molecular processes involved in tendril phyllotaxy, we carried out a RNA-Seq differential expression analysis of bulked wild-type and mutant samples of both self-pollinated and cross populations in this study. As is the case for all profiling studies, data quality is critical for drawing valid conclusions. To reduce potential false positives, we used a minimum expression threshold of at least 2 CPM for all the genes across all the RNA-Seq sample libraries in this study. The quality of the resulting data was satisfactory as clear pattern contrasts between the wild and mutant phenotypes were revealed by a MDS analysis of the expressed genes.

In this study, we found 324 DEGs, with at least 1.5-fold changes for each individual gene in the mutant and wild-type bulk samples from both self-pollinated and outcross genetic background involving the *Vitis* hybrid ‘Roger’s Red’. The high correlation coefficient (*r*=0.85) of the fold-changes of expression of these genes between the two populations suggested that these 324 genes were likely involved in the mutant tendril phenotype in the populations studied. When we examined the subsets of DEGs within the enriched GO biological processes, a substantial proportion of them were TFs and their predicted target genes. This highly suggested a substantial role these TFs may have in the observed differential expression. Indeed, consistent with early reports,^15^ the expressed genes in grapevine tendril included the AP2/ERF, NAC and WRKY TF families. These TF gene families were known to mediate plant defense response,^94^ hormone response^66^ and abiotic stress tolerance,^95^ and hence it was not a surprise that they had a large role in the resulting enriched GO terms. Molecular studies of tendrils in other species have shown that TFs likely had a significant role in tendril development. For example, the leaflet-derived tendrils of garden pea (*Pisum sativum*) was attributed to loss-of-function of the Tl gene, which encodes a Class I homeodomain leucine zipper TF.^96^ Similarly, the tendril-less melon (*Cucumis melo*) mutant, Chiba Tendril-Less (ctl), was thought to be a single-base deletion in a CmTCP1 gene (a TCP TF gene).^97^

Our DDN analysis, which examined the transcriptional ‘re-wirings’ between the mutant and normal phenotypes, provided detailed information of how various TFs might contributed to the tendril development. The condition-specific (mutant versus wild) connections among the top 10 hub genes revealed diverse and complex overlaps of several regulatory and metabolic pathway genes, which mainly were related to stress responses, plant hormones and secondary metabolites. The emerging theme from the expression and network enrichment analyses was the apparent importance of *cis*-regulatory interactions of the major stress-responsive TFs in tendril phyllotaxy. This was reflected in the fact that the combined number of downstream targets of the TFs AP2/ERF, WRKY, NAC and TCP accounted for about 38% (123 of the 324) of the total discovered DEGs. In addition, a dependency network constructed with these *cis*-regulatory connections as *a priori *information revealed that the extensively connected hub genes were either TFs or important genes in developmental pathways. More importantly, the significant re-wirings of these hub genes involved at least one (de)activated TF.

MYB12 is a flavonol-specific TF named *VvMYBF1* in grapevine.^62^ It regulates the first step in flavonol biosynthesis (that is, Flavonol Synthase 1 or FLS) and thus is a key activator of flavonol biosynthesis gene.^89^ In addition to the widely recognized role in controlling the synthesis of flavonols, *VvMYBF1* also forms a ternary complex with the TFs bHLH and WD40, referred as the MBW ternary complex, to activate the anthocyanin synthesis pathway.^89^ As anthocyanins and flavonoids are important domestication traits in grapevine related to berry and wine-making quality, the roles of *VvMYBF1* and a host of other MYB TFs and regulators in this pathway have been widely expanded to include not only pigmentation, berry ripening and cell fate, but also plant defense response, drought tolerance, pathogen resistance and light-sensing response.^98^ Interestingly, it has recently been shown that TCP3 enhanced flavonoid biosynthesis by interacting with MYB12 and thus (de)stabilizing a MBW complex.^89^ It was reported that a destabilized flavonol and anthocyanin pathway by TCP3 interaction affected the auxin biosynthesis and auxin-related processes.^99,100^ Furthermore, in *Arabidopsis*, such TCP-MYB12 interaction was shown to affect several pathways that manifested into an altered leaf phyllotaxy, abnormal vasculature patterning, reduced apical dominance, impaired root development and reduced organ size.^101^

Another TF that may significantly affect tendril phyllotaxy is ERF17. ERF17 is an AP2/ERF TF with the single-most number of DEG targets (~13% of 324 DEGs). Our co-dependency network analysis suggested that ERF17 had a significant interaction with ROXY19, which belongs to the glutaredoxin (GRX) gene family, a class of oxidative response genes. In *Arabidopsis*, two GRX genes were shown to have important roles in petal formation via post-translational modification.^71^ This finding offers an interesting avenue for further investigation, as grapevine tendril is a modified inflorescence^7,13^ expressing flower development genes.^15^ Interestingly, one of the four genes identified to be uniquely expressed in the mutant background was also an AP2/ERF TF (ERF22, GSVIVT01036388001).

In addition to the TFs discussed above, we have also found some lignin-related genes, which were likely involved in the mutant phyllotaxy observed. Among the four uniquely expressed genes we examined (only expressed in the wild-type or mutant background), we found two lignin-related genes, *PRX52* (GSVIVT01009107001) and lacasse (GSVIVT01013693001), whose expressions were, respectively, enhanced and suppressed in the mutant background. PRX52 was previously found to have a key role in lignin synthesis,^78^ whereas the lacasse gene was involved in lignin degradation.^39^ Interestingly, our dependency network analysis independently showed that the same lignin biosynthesis gene, *PRX52*, was co-regulated by two important transcription factors, TCP3 and MYB12, which were identified to have key roles in tendril phyllotaxy, as discussed earlier. These evidences collectively suggested that the genes in the lignin pathway might have contributed to the tendril phyllotaxy of the mutant observed in this study. Indeed, lignification, cell wall development and cell proliferation are processes ontologically related with shoot architecture development^102^ and likely with tendril development as well.

To conclude, tendril phyllotaxy is an important developmental trait and likely has a complex basis of genetic control. Unfortunately, we have very little knowledge about the global molecular processes of the development of the trait. Our work in this study is the first attempt to fill this knowledge gap in the literature. Through analyzing 324 DEGs from both selfed and outcrossed populations, we found several TFs, which likely had significant roles, through regulating DEGs and others, in contributing to the development of tendril phyllotaxy. TCP3, a known master integrator in growth and development, appeared to be one of the key TF genes involved in the process. Among the structural genes, we have found several lignin-related genes likely involved in tendril development. Like in many profiling studies, the results reported in this study would have to be validated in the future experiments. Nevertheless, these results should provide the first insight of the complex molecular events involved in grapevine tendril development.

## Figures and Tables

**Table 1:** RNA-Seq bulk samples and their population background, phenotypes and other properties

*Bulk sample ID*	*Population*	*Phenotype*	*No. of individual progeny vines*	*Library size (Gb)*
P65.m.2	P65—crossed	Mutant	8	2.98
P65.m.3	P65—crossed	Mutant	9	4.40
P65.m.4	P65—crossed	Mutant	11	5.93
P65.n.1	P65—crossed	Wild-type	9	4.25
P65.n.2	P65—crossed	Wild-type	9	1.67
P96.m.1	P96—selfed	Mutant	4	3.45
P96.m.2	P96—selfed	Mutant	4	2.98
P96.m.3	P96—selfed	Mutant	5	4.23
P96.n.4	P96—selfed	Wild-type	4	3.05
P96.n.5	P96—selfed	Wild-type	4	1.99

**Table 2:** Chi-square significance tests of mutant versus wild-type segregations of tendril distribution patterns in fitting the gene action model of two epistatic supplementary dominant loci in selfed (P96) and cross (P65) populations, respectively

*Phenotype*	*P96 selfed population*		*P65 cross population*	
	*Observed*	*X*^*2*^ *fit test for fitting 9 mutant:7 wild-type*	*Observed*	*X*^*2*^ *fit test for fitting 1 mutant: 3 wild-type*
Mutant	87	0.73^ns^	35	0.12^ns^
Wild-type	64		140	
Total	151		175	

Abbreviation: ns, non-significant.

**Table 3:** Significantly enriched GO biological terms revealed from 324 DEGs (FDR <0.10) and the GO term cluster assignments

*Significant GO term*	*Cluster*
Response to carbohydrate stimulus	I
Response to chitin	I
Response to organic substance	I
Regulation of response to stimulus	I
Response to endogenous stimulus	I
Response to chemical stimulus	I
Response to abiotic stimulus	I
Response to stress	I
Response to biotic stimulus	I
Response to ethylene stimulus	I
Jasmonic acid-mediated signaling pathway	II
Negative regulation of response to stimulus	II
Ethylene-mediated signaling pathway	II
Induced systemic resistance	II
Two-component signal transduction system (phosphorelay)	II
Amide transport	III
Urea transport	III
Regulation of cell communication	IV
Regulation of gene expression	IV

**Table 4:** Hub genes identified in the KDDN-generated dependency network

*Gene ID*	*Average log2 FC*	*No of connection*	Arabidopsis* annotation*	*Biological pathway/TF family*
GSVIVT01009007001	1.22	10	AtWind1	AP2/ERF TF
GSVIVT01015037001	1.64	9	AtERF17	AP2/ERF TF
GSVIVT01030219001	1.45	7	PRX52	Lignin biosynthesis
GSVIVT01033485001	−1.20	11	Une10	bHLH TF
GSVIVT01019515001	1.72	7	Unknown protein	—
GSVIVT01031462001	1.22	9	Superoxide dismutase	Ethylene biosynthesis
GSVIVT01027027001	2.13	7	Acidic endochitinase	Chitin degradation
GSVIVT01022164001	−1.25	9	d-alanyl-d-alanine carboxypeptidase, putative	Protein transport
GSVIVT01021507001	1.37	10	9-*cis*-epoxycarotenoid dioxygenase 2	ABA pathway
GSVIVT01009045001	−1.03	8	RNA-binding protein	Post-transcriptional regulation

**Table 5:** Most significantly enriched transcription factors among the 324 DEGs identified in this study

*Gene ID*	*Total DEGs*	*No. of targets*	*FDR*	*Annotation (*Arabidopsis* best hit)*	*TF family*
GSVIVT01015037001	324	44	5.06E−08	AtERF17	AP2/ERF
GSVIVT01018165001	324	20	1.14E−04	Une 10	bHLH
GSVIVT01009007001	324	23	4.53E−04	AtWIND1	AP2/ERF
GSVIVT01014236001	324	27	9.79E−04	TCP3	TCP
GSVIVT01012682001	324	10	4.33E−03	WRKY6	WRKY
GSVIVT01027069001	324	10	7.15E−03	WRKY30	WRKY
GSVIVT01021098001	324	23	7.94E−02	ERF9	AP2/ERF
GSVIVT01008839001	324	7	8.69E−02	AtAF1	NAC
GSVIVT01014403001	324	5	1.05E−01	RD26	NAC
