# Supplementary material for: A transcriptome analysis of two grapevine populations segregating for tendril phyllotaxy
Source: Hortic Res. 2017 Jul 12;4:17032–. doi: 10.1038/hortres.2017.32 (PMC5506248; doi:10.1038/hortres.2017.32)
Supplement: Supplementary Figure 1 [file hortres201732-s1.docx]

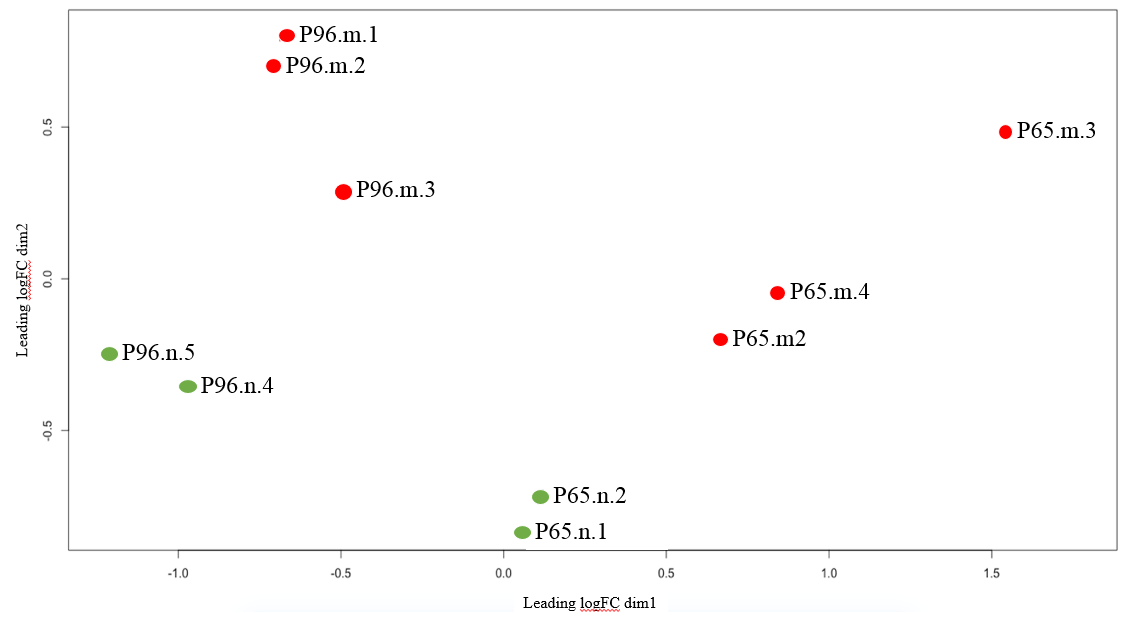


Supplementary Figure 1. A MDS plot based on the expression profiles of 13 375 genes, each with at least 2CPM in all the bulked RNA-Seq libraries.
