# Supplementary material for: A transcriptome analysis of two grapevine populations segregating for tendril phyllotaxy
Source: Hortic Res. 2017 Jul 12;4:17032–. doi: 10.1038/hortres.2017.32 (PMC5506248; doi:10.1038/hortres.2017.32)
Supplement: Supplementary Figure 2 [file hortres201732-s2.docx]

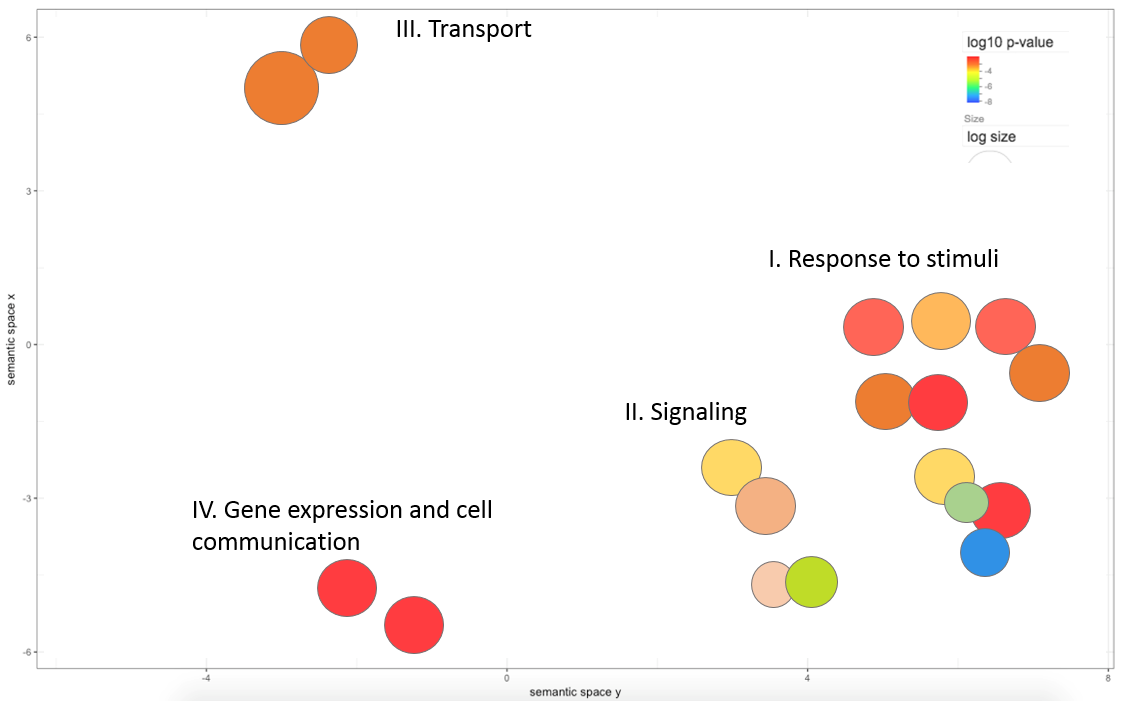


Supplemental Figure 2. A MDS plot representing 19 enriched GO biological processes and their relative similarity among the 324 DEGs.
