# Supplementary material for: A transcriptome analysis of two grapevine populations segregating for tendril phyllotaxy
Source: Hortic Res. 2017 Jul 12;4:17032–. doi: 10.1038/hortres.2017.32 (PMC5506248; doi:10.1038/hortres.2017.32)
Supplement: Supplementary Figure 3 [file hortres201732-s3.docx]

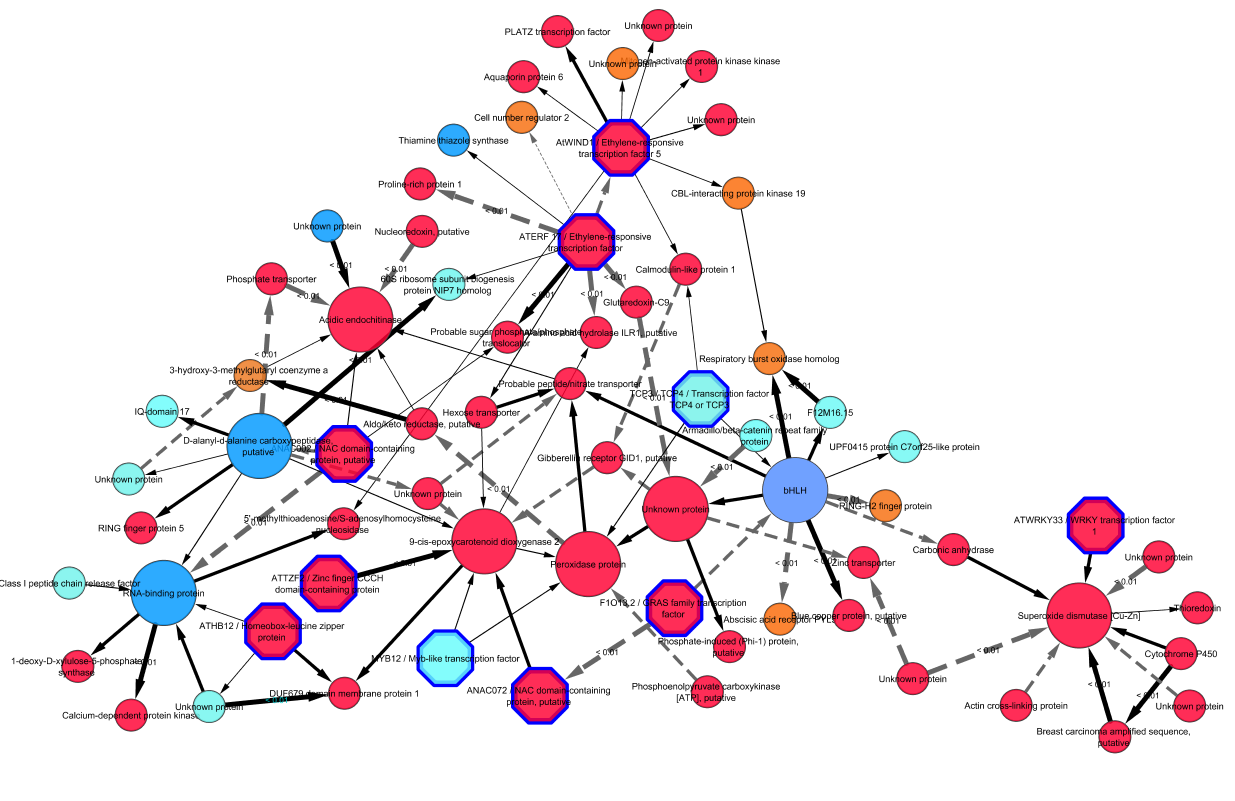


Supplemental Figure 3. A KDDN-generated dependency network among the ten most connected hub genes observed in this study. Orange and red nodes are moderately to highly up-regulated genes. Light blue to dark blue nodes are moderately to highly down-regulated genes. Octagon-shaped nodes are transcription factors. Connecting lines represent directional co-dependency in expression, with thicker lines indicate highly significant (pval < 0.01) connection in the mutant background, while broken lines indicate significantly deactivated in the mutant background. The size of a node is proportional to the number of connecting lines involved.
